# Supplementary material for: Rapid green leaf volatile sensing allows intact plants to release induced volatiles earlier than wounded emitters
Source: Proc Natl Acad Sci U S A. 2026 Oct 28;123(44):e2604047123. doi: 10.1073/pnas.2604047123 (PMC13732224; doi:10.1073/pnas.2604047123)
Supplement: Supplementary file 1 [file pnas.2604047123.sapp.pdf]

PNAS

Rapid green leaf volatile sensing allows intact plants to release induced volatiles earlier than wounded emitters

Tristan M. Cofer[1*], Lingfei Hu[1,2], Gaetan Glauser[3] Matthias Erb[1*]

[1] Institute of Plant Sciences, University of Bern, Switzerland

[2] Hainan Institute, Zhejiang University, Yazhou District, Sanya, 572025, China

[3] Neuchâtel Platform of Analytical Chemistry, University of Neuchâtel, Switzerland

*Correspondence: tristan.cofer@unibe.ch (TMC); matthias.erb@unibe.ch (ME)

This PDF file includes:

Supplementary Figure S1 to S9
Supplementary Table S1

Figures

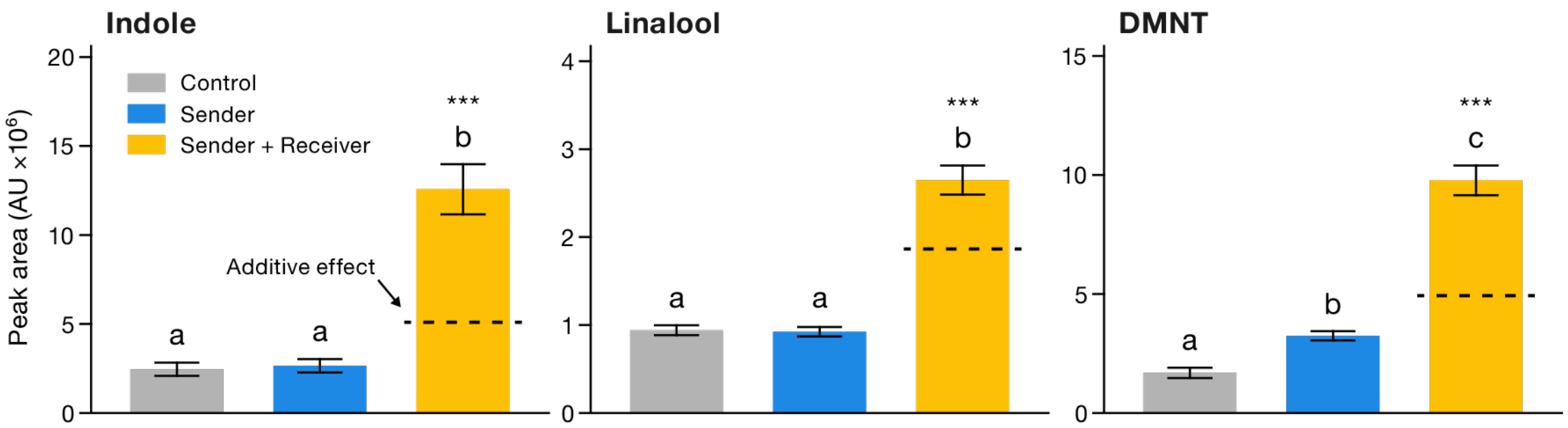

Fig. S1. Early volatile emissions from sender-receiver pairs measured by GC-MS. Emissions of indole, linalool, and DMNT measured between 60–75 min after the first induction treatment from untreated control plants, herbivore-induced sender plants, and sender plants paired with undamaged receiver plants. Headspace volatiles were collected during this interval using SuperQ adsorbent traps and analyzed by gas chromatography-mass spectrometry (GC-MS). Data are presented as mean ± SE (n = 7). Different letters indicate significant differences among treatments, as determined by one-way ANOVA followed by Tukey's honestly significant difference (HSD) test. Dashed lines represent the predicted additive emissions from herbivore-induced sender plants and untreated control plants. Asterisks indicate significant differences between sender–receiver pairs and the predicted additive values (****P* < 0.001).

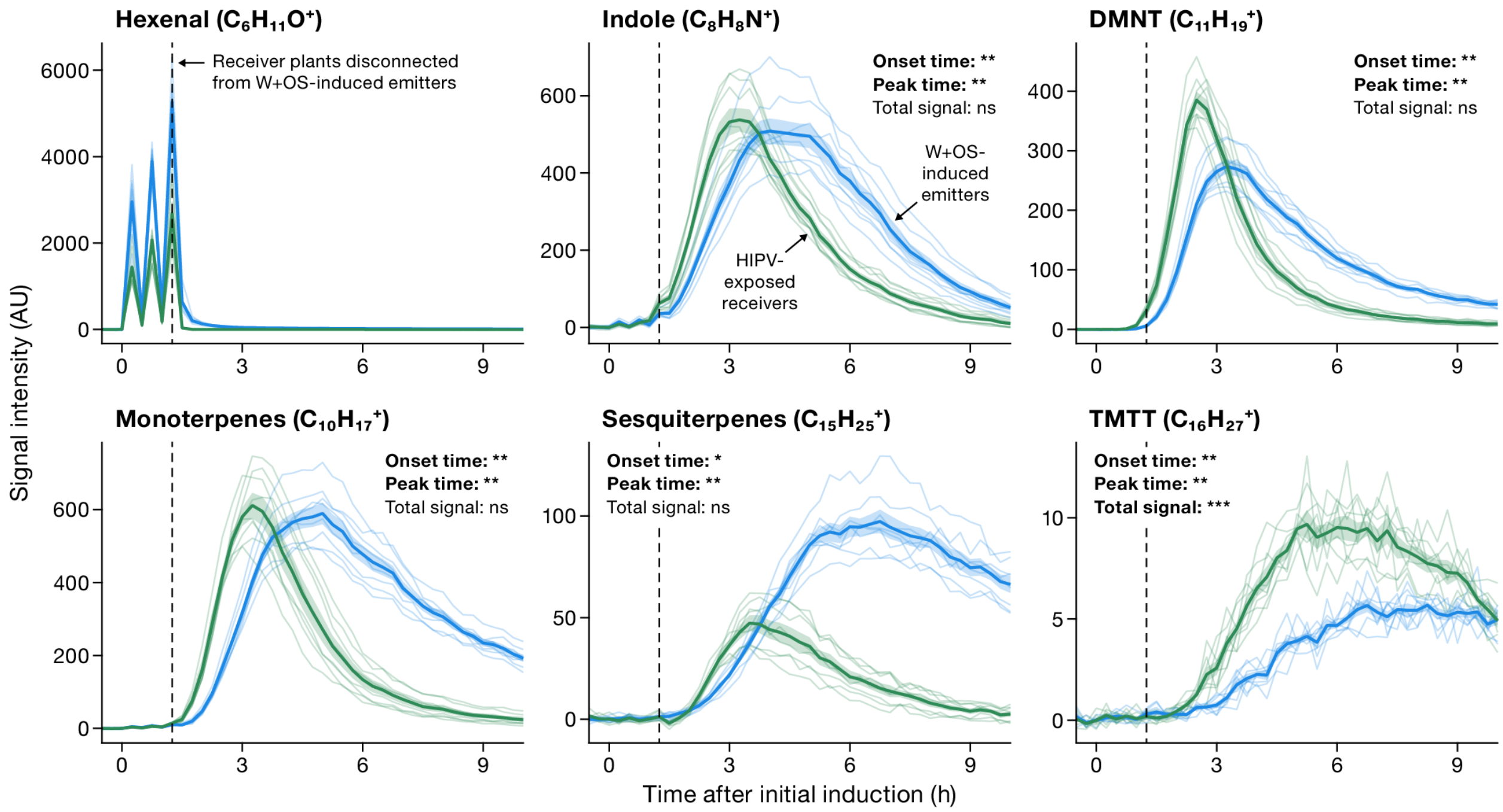

Fig. S2. Earlier volatile emissions from receivers disconnected from senders after 75 min. Sender plants were induced by mechanical wounding followed by application of insect oral secretions at 0, 30, and 60 min. Receiver plants were exposed to sender emissions from 0 to 75 min and then disconnected from the senders (dashed line). Volatile emissions from W+OS-induced emitters (blue) HIPV-exposed, disconnected receivers (green) were monitored individually using PTR-ToF-MS. Thin lines show individual biological replicates; thick lines represent mean emission rates; shaded ribbons indicate ± SEM (n = 8). For each analyte, onset time, peak time, and cumulative signal intensity were quantified from baseline-corrected time courses as described for Figure 1. Sender and receiver values were compared using two-sided Wilcoxon rank-sum tests, with Benjamini-Hochberg correction across analytes within each metric. Asterisks indicate adjusted P values ($P < 0.05$; * $P < 0.01$; ** $P < 0.001$); ns, not significant.

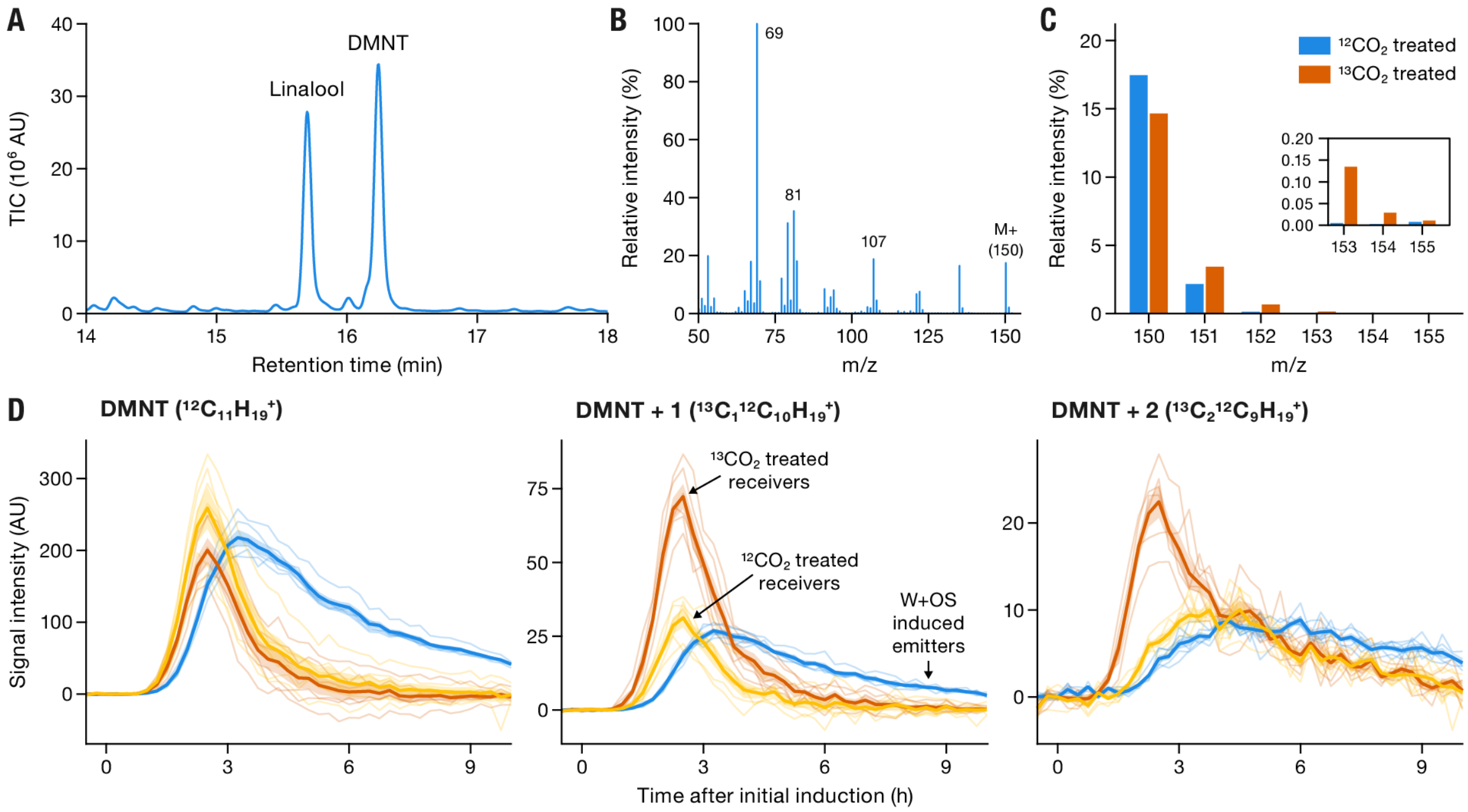

Fig. S3. $^{13}CO_2$ labeling shows that receivers release *de novo* produced DMNT earlier than senders. (A) Representative GC-MS total ion chromatogram from a $^{12}CO_2$-treated plant following W+OS induction, showing linalool and DMNT peaks. (B) Representative electron ionization mass spectrum of the DMNT peak. (C) Relative intensities of the molecular ion isotopologues of DMNT in W+OS-induced plants pretreated with $^{12}CO_2$ or $^{13}CO_2$; the inset enlarges the low-abundance *m/z* 153–155 ions. While significant incorporation of $^{13}CO_2$ was detected for DMNT, no clear incorporation was found for linalool or other induced volatiles from the pulse labelling treatment. (D) Sender plants were induced by mechanical wounding followed by application of insect oral secretions at 0, 30, and 60 min and paired with receiver plants that had been pulse-labeled with $^{13}CO_2$ (orange) or $^{12}CO_2$ (yellow). Emissions of unlabeled DMNT and the +1 and +2 ^{13}C isotopologues were monitored by PTR-ToF-MS. Sender emissions are shown in blue. Receiver emissions were calculated by subtracting mean sender emissions from emissions of sender-receiver pairs at each time point. Note that the later rising curves of the $^{13}C_2{}^{12}C_9H_{19}^+$ signal (*m/z* 153.143) in the right panel are due to an insufficiently resolved, closely related m/z signal from another compound (m/z 153.128). Thin lines show individual biological replicates; thick lines represent mean emission rates; shaded ribbons indicate ± SEM (n = 6).

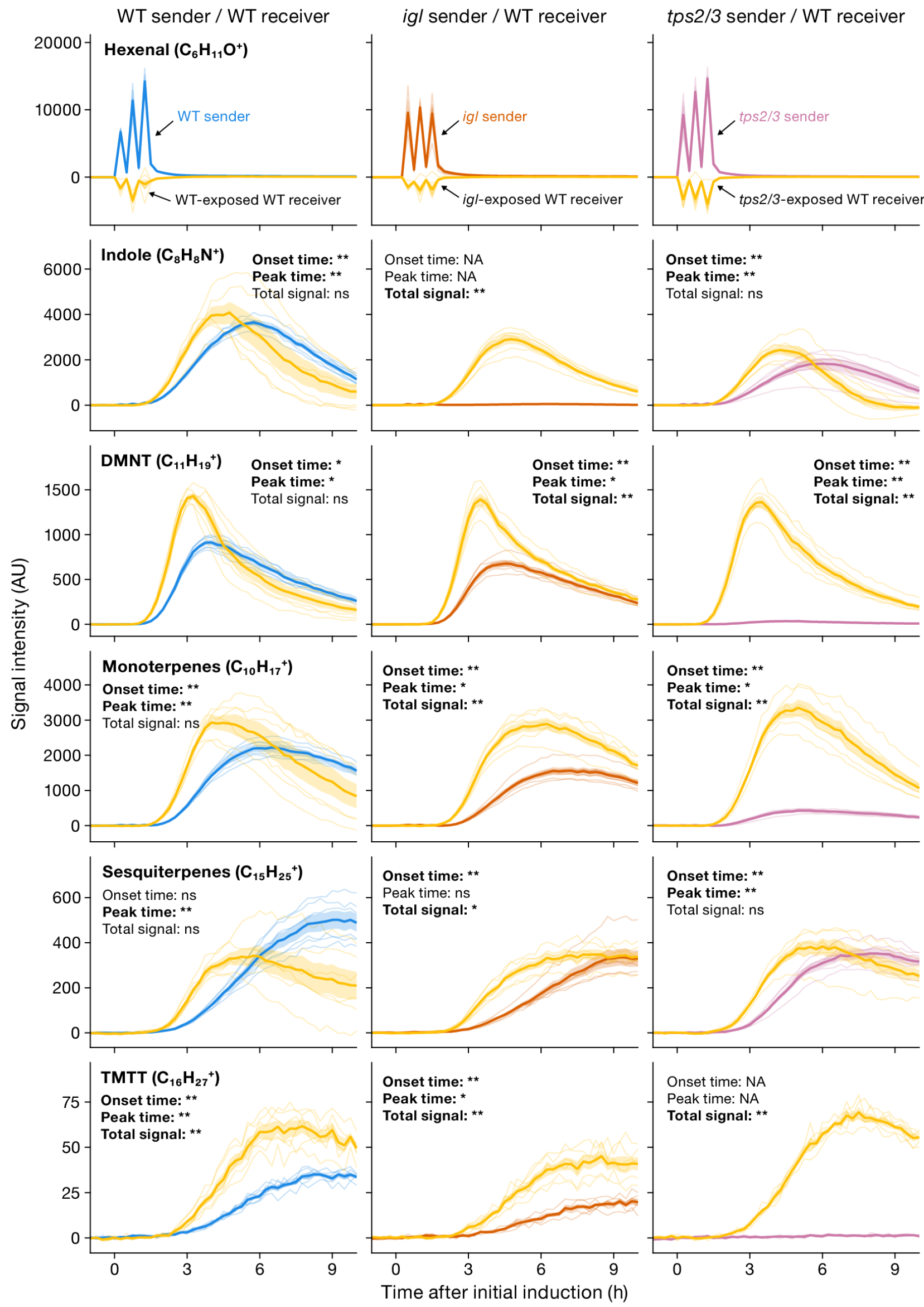

Fig. S4. Indole, mono- and homoterpenes are not required for rapid responses in neighboring plants. Time-resolved volatile emissions were measured from wild-type (WT) receiver plants exposed to herbivory-induced WT or mutant sender plants carrying defects in major volatile biosynthesis pathways. Sender plants were induced by mechanical wounding followed by application of insect oral secretions at 0, 30, and 60 min, and volatile emissions were monitored every 15 min from sender plants alone and from sender-receiver pairs. Receiver emissions were calculated by subtracting emissions from sender plants alone from those measured in sender-receiver pairs. Shown are emissions from WT, *igl*, and *tps2/3* sender plants paired with WT receivers. The *igl* mutant is deficient in indole biosynthesis, whereas *tps2/3* is impaired in mono- and homoterpene biosynthesis. Stress-related volatiles, including (*Z*)-3- and (*E*)-2-hexenal (combined signal), indole, mono-, sesqui-, and homoterpenes (DMNT, TMTT), were detected as protonated ions based on characteristic *m/z* signals. Solid lines represent mean emission rates for sender plants (blue, orange, or pink) and WT receiver plants (yellow); shaded ribbons indicate ± SEM, and thin lines show individual biological replicates (n = 6). For each analyte, onset time, peak time, and cumulative signal intensity were quantified from baseline-normalized time courses. Statistical differences were assessed using two-sided Wilcoxon rank-sum tests with Benjamini-Hochberg correction for multiple comparisons. Asterisks indicate significance levels (*P < 0.05; **P < 0.01); ns, not significant; NA, metric not determined. For other mutants, see Fig. 4.

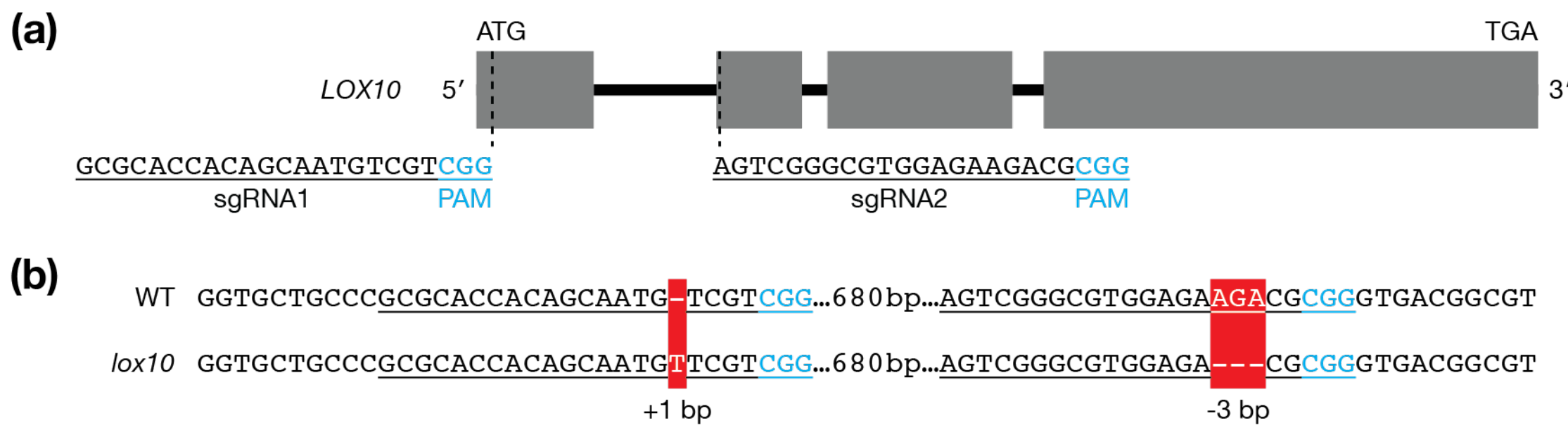

Fig. S5. (a) Schematic representation of the *LOX10* gene structure showing exon-intron organization (gray boxes, exons; black lines, introns). Two single guide RNAs (sgRNA1 and sgRNA2) were designed to target the 5′ region of the coding sequence to maximize the likelihood of generating a loss-of-function allele. The protospacer-adjacent motif (PAM) sequences are indicated in blue. (b) Sequence alignment of the targeted regions in wild type (WT) and the *lox10* mutant allele. Red boxes indicate mutations at the sgRNA target sites relative to WT. A one-base insertion (+1 bp) was detected at the first target site (sgRNA1), and a three-base deletion (−3 bp) was identified at the second target site (sgRNA2). PAM sequences are highlighted in blue and target sequences are underlined. These mutations are predicted to disrupt *LOX10* function.

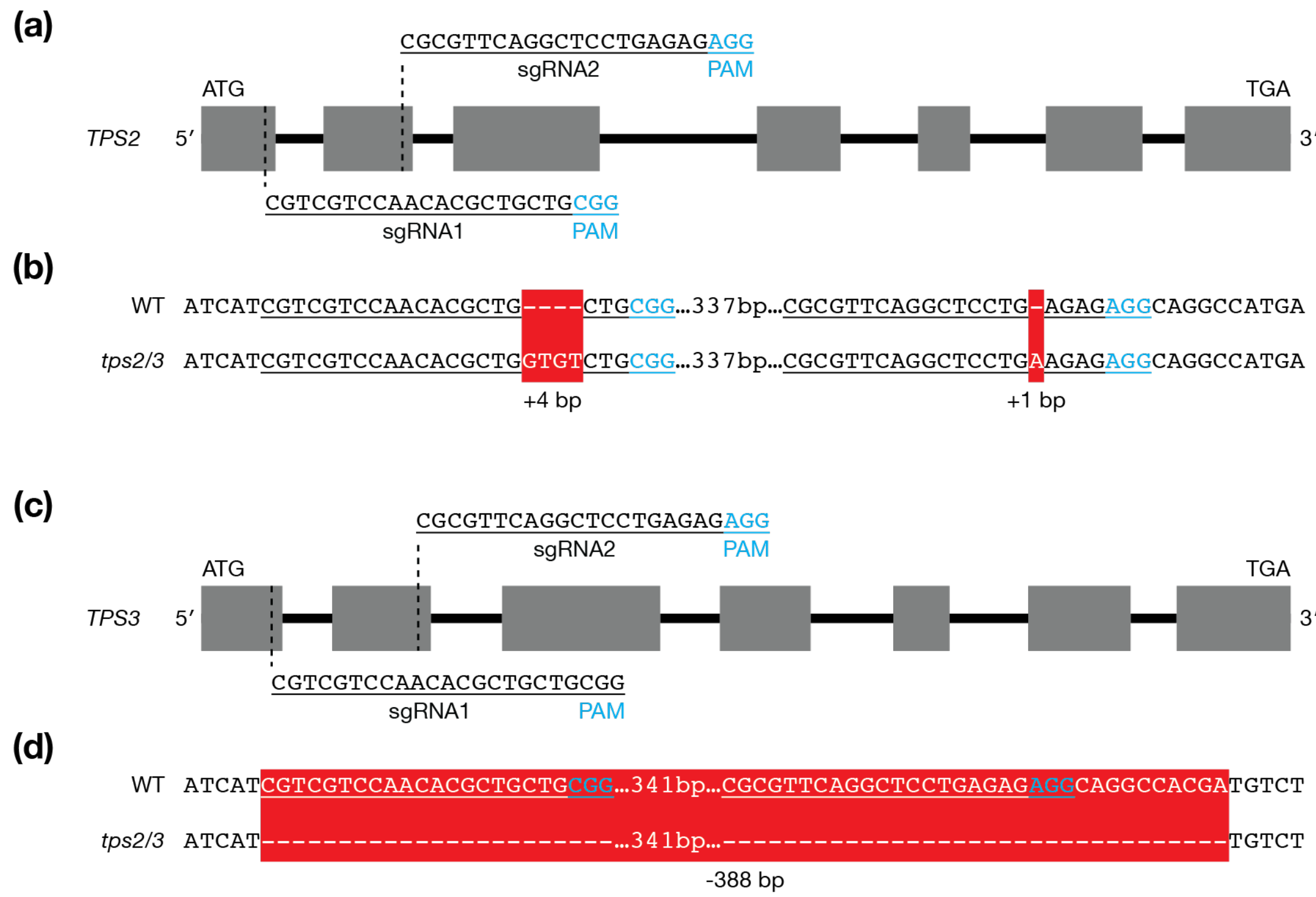

Fig. S6. (a) Schematic representation of the *TPS2* gene structure showing exon-intron organization (gray boxes, exons; black lines, introns). Two single guide RNAs (sgRNA1 and sgRNA2) targeting conserved coding regions near the 5′ end were designed. The protospacer adjacent motif (PAM) sequences are indicated in blue. (b) Sequence alignment of the targeted regions in wild type (WT) and the *tps2/3* mutant for *TPS2*. Red boxes indicate insertions at the sgRNA1 (+4 bp) and sgRNA2 (+1 bp) target sites relative to WT. PAM sequences are highlighted in blue and target sequences are underlined. The mutations result in frameshifts predicted to disrupt *TPS2* function. (c) Schematic representation of the *TPS3* gene structure with the same sgRNA1 and sgRNA2 target sites as used for *TPS2*. Exons and introns are indicated as in (a), and PAM sequences are shown in blue. (d) Sequence alignment of the targeted regions in WT and *tps2/3* for *TPS3*. A large deletion of 388 bp between the two sgRNA target sites was detected in the mutant allele. The deleted region (red box) spans both target sites, leading to a frameshift and predicted loss of *TPS3* function.

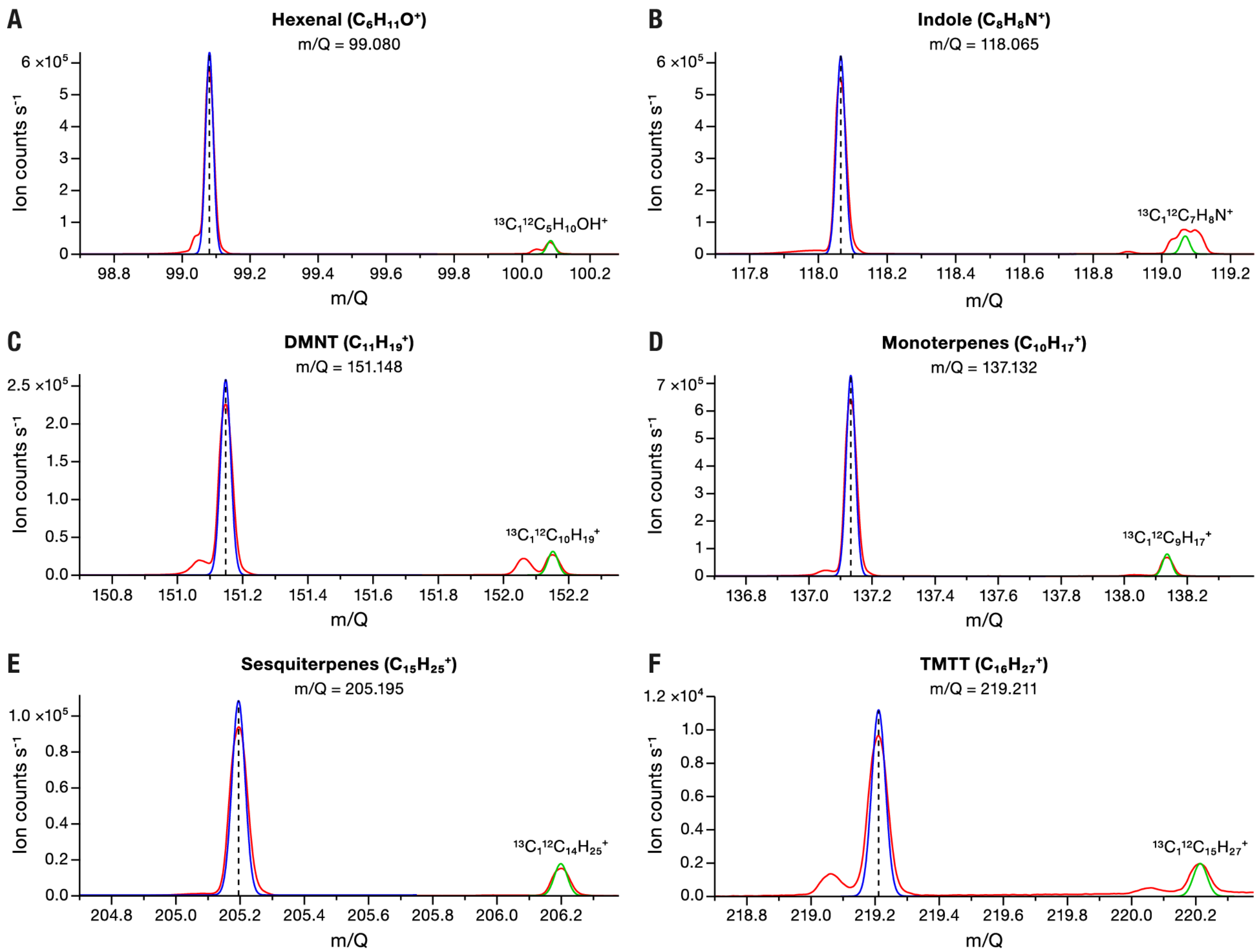

Fig. S7. High-resolution PTR-ToF-MS spectra support assignment of monitored volatile ions. Mass spectra are shown for (A) hexenal, (B) indole, (C) DMNT, (D) monoterpenes, (E) sesquiterpenes, and (F) TMTT. The expected monoisotopic ion is marked by a dashed line, and the corresponding ^{13}C isotopologue is shown at the expected higher m/Q.

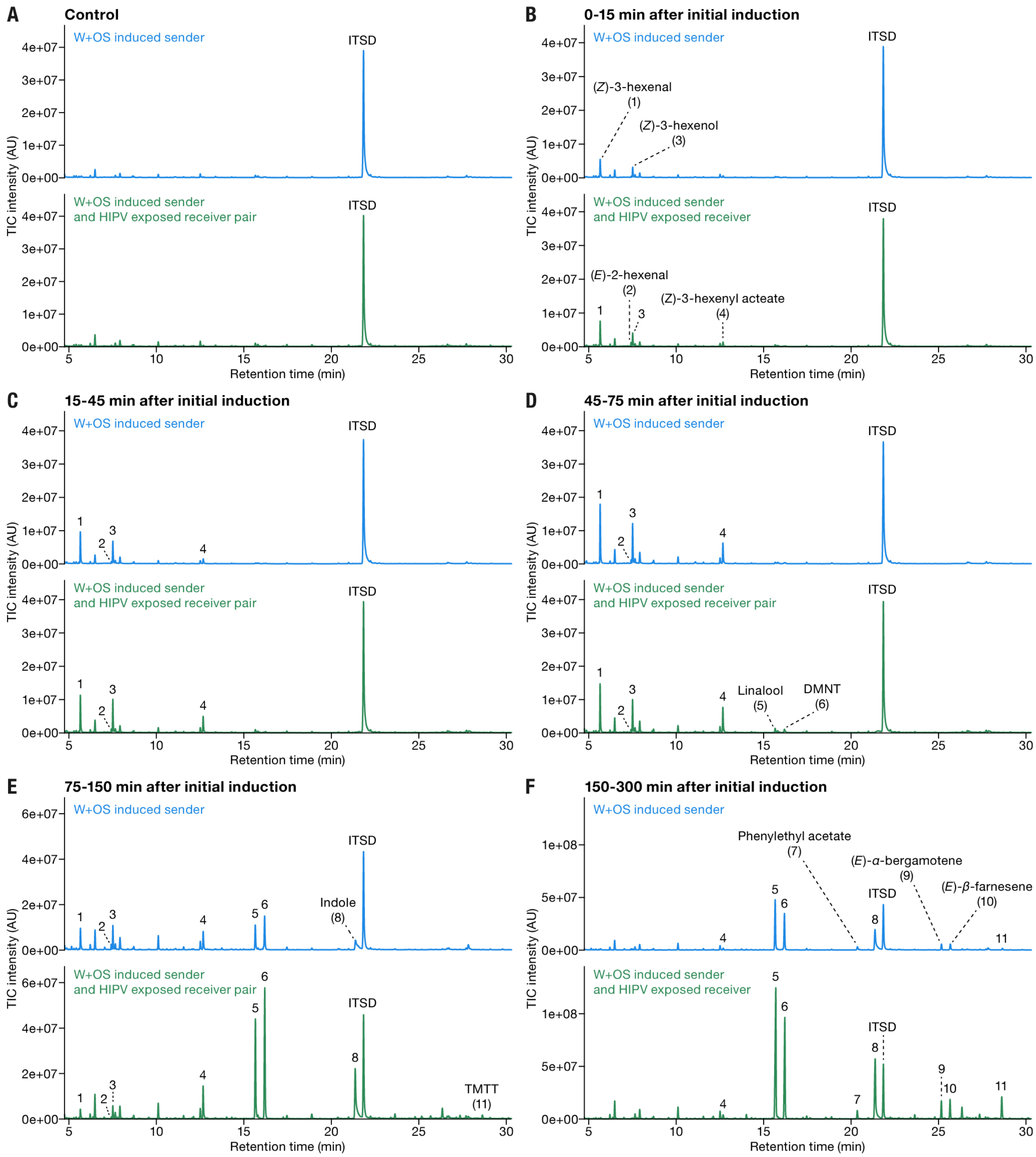

Fig. S8. GC-MS chromatograms of volatile emissions from W+OS-induced sender plants and sender-receiver pairs. Sender plants were induced by mechanical wounding followed by application of insect oral secretions at 0, 30, and 60 min. Representative total ion chromatograms of SuperQ-collected headspace samples are shown for untreated controls (A) and samples collected 0–15 min (B), 15–45 min (C), 45–75 min (D), 75–150 min (E), and 150–300 min (F) after initial induction. Sender plants alone are shown in blue, and sender plants paired with HIPV-exposed receivers are shown in green. Identified peaks are (1) (*Z*)-3-hexenal, (2) (*E*)-2-hexenal, (3) (*Z*)-3-hexenol, (4) (*Z*)-3-hexenyl acetate, (5) linalool, (6) DMNT, (7) phenylethyl acetate, (8) indole, (9) (*E*)-*α*-bergamotene, (10) (*E*)-*β*-farnesene, and (11) TMTT. ITSD, nonyl acetate internal standard.

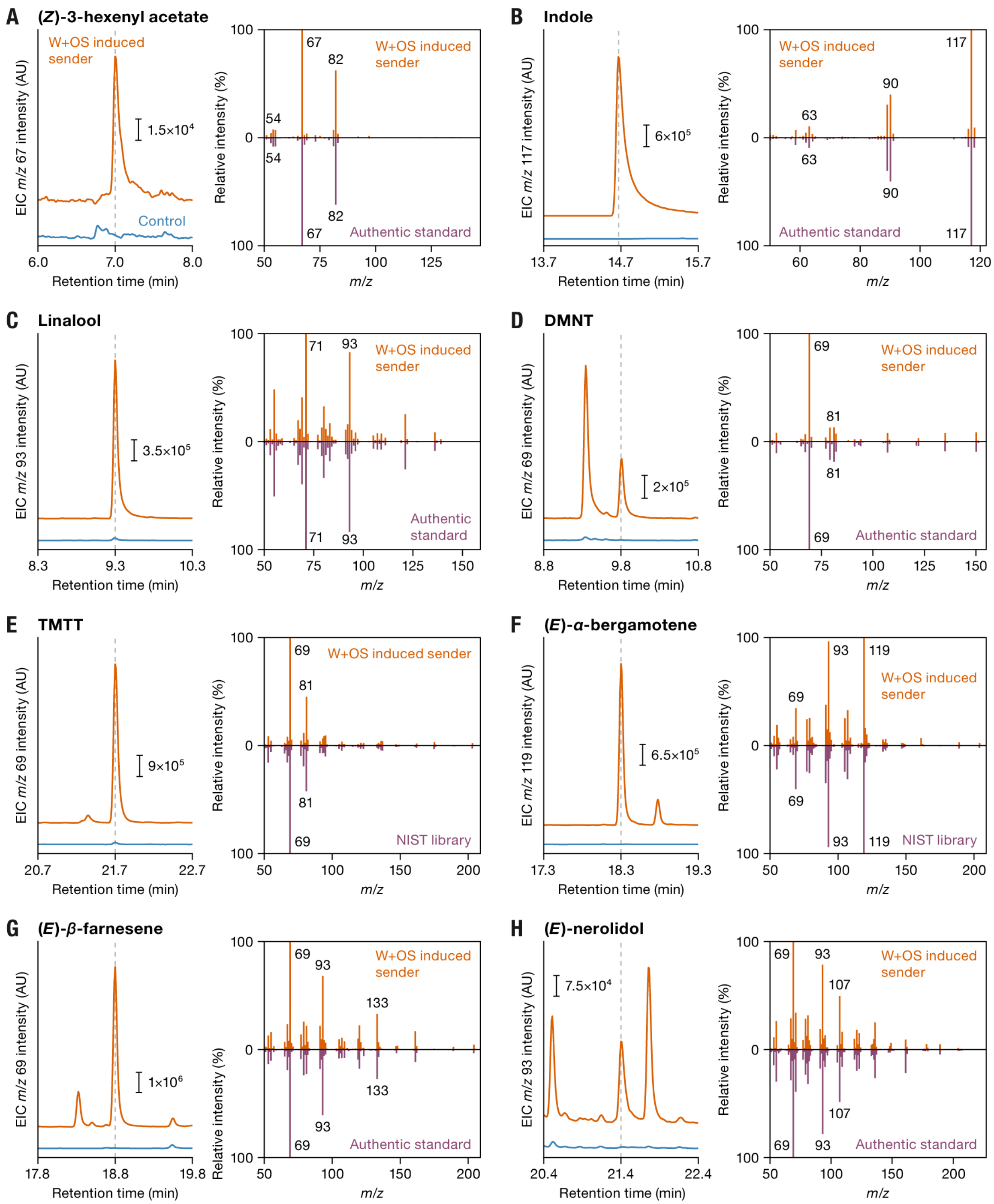

Fig. S9. SPME-GC-MS validation of within-leaf volatile compound assignments. Representative extracted ion chromatograms from untreated control leaves (blue) and W+OS-induced sender leaves (orange), together with their corresponding electron ionization mass spectra. Mirrored spectra show authentic standards (purple) for (A) (*Z*)-3-hexenyl acetate, (B) indole, (C) linalool, (D) DMNT, (G) (*E*)-*β*-farnesene, and (H) (*E*)-nerolidol, and NIST library spectra for (E) TMTT and (F) (*E*)-*α*-bergamotene. Dashed lines indicate the retention time of each assigned compound.

Tables

Table S1. Primers used in this study.

Name	Sequence (5′–3′)
IGL-Fw	GAGCTGGTGCTGCTCACA
IGL-Rv	GCGCGTGGACCTGTAACT
FPPS3-Fw	CCTGGCTAGTTGTGCAAGCT
FPPS3-Rv	CAAAACAGTTTGGACTGCCT
TPS2-Fw	TACCGGGTCGAGATCACCAA
TPS2-Rv	TCGTTCGTAACGGTGTGGAG
TPS3-Fw	CAAGGAGCATGGATCTAACCCTA
TPS3-Rv	AAGCAGACCTGCGCGATGTC
TPS10-Fw	TGACAGCCTTGATCACCGTA
TPS10-Rv	AGTTCATCCCCACACGAATC
CYP92C5-Fw	AGGGGTTCAAGCGGAAGATG
CYP92C5-Rv	AGGTCAGTCGCCACAAACTC
ACTIN1-Fw	GGGATTGCCGATCGTATGAG
ACTIN1-Rv	GAGCCACCGATCCAGACACT
